# Neural bases for the genesis and CO_2_ therapy of periodic Cheyne–Stokes breathing in neonatal male connexin-36 knockout mice

**DOI:** 10.3389/fnins.2023.1045269

**Published:** 2023-02-08

**Authors:** Ana M. Casarrubios, Leonel F. Pérez-Atencio, Cristina Martín, José M. Ibarz, Eva Mañas, David L. Paul, Luis C. Barrio

**Affiliations:** ^1^Units of Experimental Neurology and Sleep Apnea, Hospital “Ramón y Cajal” (IRYCIS), Madrid, Spain; ^2^Ph.D. Program in Neuroscience, Autonoma de Madrid University-Cajal Institute, Madrid, Spain; ^3^Sleep Apnea Unit, Respiratory Department, Hospital “Ramón y Cajal” (IRYCIS), Madrid, Spain; ^4^Department of Neurobiology, Medical School, Harvard University, Boston, MA, United States; ^5^Center for Biomedical Technology, Universidad Politécnica de Madrid, Madrid, Spain

**Keywords:** central sleep apnea, periodic breathing, respiratory oscillators, coupled oscillators, carbon dioxide

## Abstract

Periodic Cheyne–Stokes breathing (CSB) oscillating between apnea and crescendo–decrescendo hyperpnea is the most common central apnea. Currently, there is no proven therapy for CSB, probably because the fundamental pathophysiological question of how the respiratory center generates this form of breathing instability is still unresolved. Therefore, we aimed to determine the respiratory motor pattern of CSB resulting from the interaction of inspiratory and expiratory oscillators and identify the neural mechanism responsible for breathing regularization induced by the supplemental CO_2_ administration. Analysis of the inspiratory and expiratory motor pattern in a transgenic mouse model lacking connexin-36 electrical synapses, the neonatal (P14) Cx36 knockout male mouse, with a persistent CSB, revealed that the reconfigurations recurrent between apnea and hyperpnea and vice versa result from cyclical turn on/off of active expiration driven by the expiratory oscillator, which acts as a master pacemaker of respiration and entrains the inspiratory oscillator to restore ventilation. The results also showed that the suppression of CSB by supplemental 12% CO_2_ in inhaled air is due to the stabilization of coupling between expiratory and inspiratory oscillators, which causes the regularization of respiration. CSB rebooted after washout of CO_2_ excess when the inspiratory activity depressed again profoundly, indicating that the disability of the inspiratory oscillator to sustain ventilation is the triggering factor of CSB. Under these circumstances, the expiratory oscillator activated by the cyclic increase of CO_2_ behaves as an “anti-apnea” center generating the crescendo–decrescendo hyperpnea and periodic breathing. The neurogenic mechanism of CSB identified highlights the plasticity of the two-oscillator system in the neural control of respiration and provides a rationale base for CO_2_ therapy.

## Introduction

Periodic Cheyne–Stokes breathing (CSB), characterized by an oscillating pattern between apnea/hypopnea and crescendo–decrescendo hyperpnea (Cheyne, 1818; Stokes, 1854), is perhaps the most common form of central sleep apneas (AASM, 2014). The etiology of CSB is heterogeneously raising the question of whether CSB is an adaptive compensatory mechanism or detrimental pathological respiration. CSB commonly occurs in premature and term infants during the first few weeks of life (Fenner et al., 1973) and in healthy adults at high altitudes (Ainslie et al., 2013). CSB is also of clinical concern as found in a third of patients with heart failure associated with a worse prognosis (Terziyski and Draganova, 2018) and a quarter of patients with acute ischemic stroke (Siccoli et al., 2008), as well as in patients with complex sleep apnea (Javaheri et al., 2009), in chronic opioid use (Walker et al., 2007), and idiopathic central sleep apnea syndrome (Xie et al., 1995). Mechanistically, it is well established that self-sustained periodic breathing results from large oscillations of CO_2_ around the hypocapnia-induced apneic threshold (Berssenbrugge et al., 1983; Giannoni et al., 2010) and that this propensity of CO_2_ to oscillate is paradoxically a consequence of exacerbated chemoreflex to CO_2_, in which the “loop gain” of feedback control exceeds unity resulting in a higher response than prior disturbance (Khoo et al., 1982; Sands et al., 2016). Nonetheless, it is unknown how the respiratory center generates the cyclic restart of crescendo–decrescendo hyperpnea. The respiratory rhythmicity arises from the coordinated interaction of two anatomically and functionally distinct oscillators in the brainstem: an inspiratory oscillator at the preBötzinger complex (preBötC), the kernel of respiration that drives inspiratory musculature (Smith et al., 1991; Tan et al., 2008), and a conditional expiratory oscillator in the lateral parafacial group (pF_*L*_; also termed parafacial respiratory group, pFRG), driving active expiration in states of elevated metabolic demand (Mellen et al., 2003; Onimaru and Homma, 2003; Janczewski and Feldman, 2006; Abdala et al., 2009; Huckstepp et al., 2015). This two-oscillator system establishes during early embryonic development, varying its hierarchical organization during pre-and postnatal maturation and the degree of activation of each one with the metabolic and physiological states (e.g., rest/exercise or sleep/wake) (Oku et al., 2007; Thoby-Brisson et al., 2009; Pagliardini et al., 2011; Andrews and Pagliardini, 2015; Huckstepp et al., 2016; Leirão et al., 2018). However, a fundamental unresolved question is how this two-oscillator system can generate periodic breathing. In this regard, we postulate that the activation of expiratory musculature driven by the expiratory oscillator during hypercapnia (Janczewski and Feldman, 2006; Abdala et al., 2009; Leirão et al., 2018) would play a critical role in the genesis of CSB. In a prior study, we report breathing instability in the transgenic mice lacking connexin-36 electrical synapses within respiratory networks at postnatal day fourteen (P14) that can take the form of persistent periodic respiration in a subset (14%) of male Cx36 knockout mice (Belluardo et al., 2000; Deans et al., 2001; Solomon, 2003; Pérez-Atencio et al., 2021). This animal model is the stage for the current study addressing the neural basis of CSB. First, we compared the characteristics of periodic breathing in Cx36KO mice with those of CSB in humans to validate our animal model as a suitable model of CSB. Next, we identified the inspiratory and expiratory motor patterns underlying CSB. Finally, we elucidated the neural mechanism responsible for the suppression of CBS and regularization of respiration with the therapy of supplemental CO_2_ administration (Berssenbrugge et al., 1983; Xie et al., 1997; Giannoni et al., 2010; Sands et al., 2016).

## Materials and methods

### Animals

Fifteen male homozygous connexin-36 knockout mice (Cx36−/−, C57BL/6; Deans et al., 2001) at postnatal day 14 (P14) were used in this study. Female Cx36KO mice were excluded because they rarely showed periodic breathing. The Local Ethics Committee approved animal handling and experimentation protocols (PROEX 165/16), according to the application of European 86/609 and Spanish 1201/2005 laws.

### Patient data

Polysomnographic data from patients with CSB were provided by the Sleep Apnea Unit of Ramón and Cajal Hospital under written informed consent.

### Experimental design

Conscious mice were placed in a face-down position in a gaseous exchange chamber (6 L) with a constant airflow (6 L/min); mice were partially immobilized by extremities with adhesive tape to preserve the stability of recordings. After 30 min habituation with medicinal air, animals were exposed to a series of near square-wake gas challenges of hypercapnia (4, 8, and 12% CO_2_ with 21% O_2_ and supplemented with N_2_), hypoxia (8% O_2_ and 92% N_2_), and hypoxia–hypercapnia (8% O_2_, 12% CO_2_, and 80% N_2_). Each stimulus was of 10 min duration and followed by periods of at least 30 min with medicinal air for full-recovery cardiorespiratory basal status.

### Electrophysiological recording

During these experimental procedures, EMG activity of inspiratory and expiratory musculatures, thoracic respiratory excursions, arterial saturation of O_2_, and partial pressure of CO_2_ were continuously monitored. Insulated bipolar platinum–iridium hook electrodes (1–1.5 MΩ) except for the active tip were implanted under anesthesia with 2% isoflurane in 100% O_2_ at the abdominal surface of the diaphragm (*EMG*_*D*_), the ninth interosseous space for recording inspiratory external and expiratory internal intercostal muscles (*EI* and *II* in *EMG*_*I*_) and the oblique abdominal muscles (*EMG*_*Abd*_). Then, animals received analgesia, and they fully recovered after anesthesia in 30–60 min. Respiratory chest wall motion (*Vent*), proportional to the volume of air inhaled and exhaled detected by a thermistor flowmeter, was measured using a homemade device based on Hall’s effect with a magnet and a magnetic sensor on each side of the thorax. Pulse oximetry with a neck collar was used to measure arterial saturation of O_2_ (*S_*a*_O_2_*; MouseOx, Starr Life Science), and the partial pressure of CO_2_ (*PtcCO*_2_) was measured with a non-invasive transcutaneous sensor placed on the abdomen skin (V-sign Sensor 2, SenTec). *S_*a*_O_2_* and *P_*tc*_CO_2_* calibrations were obtained from a blood sample of the tail artery.

### Data processing

AC signals of chest wall motion and EMG were amplified (gain ×10 and ×1,000), filtered (5–500 Hz and 100–2,000 Hz), sampled at 5 kHz (Biomedical Workbench), and then processed offline with Spike2, R, and MATLAB software. Raw EMG activities were full-wave rectified and integrated with a time constant of 5 ms (*∫EMG_*D*_, ∫EMG_*I*_, ∫EMG_*Abd*_*). The parametrization cycle-by-cycle of the expiratory–inspiratory coupling (Figure 3, *inset*) was obtained by measuring the peak–valley amplitude of ventilatory motion (*AV*), the peak intensity of expiratory and inspiratory activities (*IA_*D*_, IA_*EI*,_ IA_*II*_*), and the slope of inspiratory ramps (*SI_*D*_, SI_*EI*_*,) from the fully wave rectified EMG signals, the period of breathing cycle (*BP*), the time of the inspiration (*TI*), and the time of expiration (*TE*), which was divided into three phases, a passive postinspiratory expiration, an active expiration and a passive preinspiratory expiration (*TE_1_, TE_2_*, and *TE*_3_).

**FIGURE 1:**
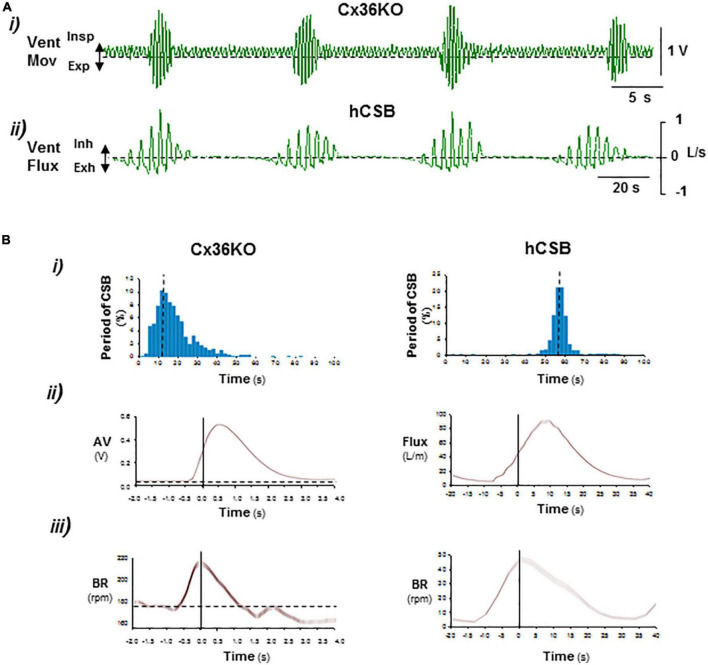
Comparison of periodic respiration of postnatal connexin-36 knockout mouse with human Cheyne–Stokes breathing. **(A)** Representative records of ventilatory movement in P14 Cx36KO mouse (*Vent Mov)* and ventilatory flux (*Vent Flux*) in human Cheyne–Stokes breathing, hCSB (i,ii). **(B)** Respiratory parameters of breathing patterns shown in **(A)** (*N* = 30 CSB cycles for Cx36KO and hCSB). (i) Interval histogram of oscillatory period, (ii) average of ventilatory motion amplitude in Cx36KO (*AV*) and flux in hCBS, and (iii) breathing rate (*BR*). Periodic breathing of the Cx36KO mouse shares all pathognomonic features of hCSB but on a faster time scale.

**FIGURE 2:**
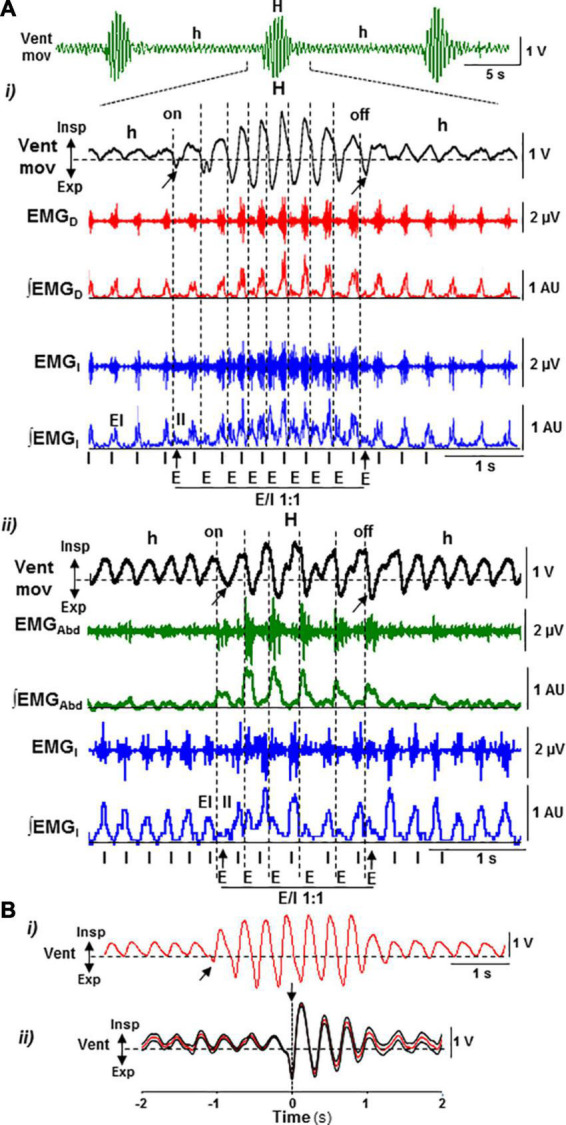
Motor pattern of periodic Cheyne–Stokes breathing in the postnatal Cx36KO mouse model. **(A)** CSB motor pattern underlying recurrent epochs of hypopnea and hyperpnea (*Vent Mov*, *h*, and *H*) oscillates between an inspiratory driven rhythm (*I*) of low intensity and synchronous in the diaphragm (*EMG_*D*_, ∫EMG_*D*_*) and external intercostal muscles (*EMG_*I*_, ∫EMG_*I*_, EI*) during hypopnea epochs and a dual expiratory (*E*) and inspiratory (*I*) motor activity coupled in an antiphase ratio 1:1 (*E/I 1:1*), due to the additional expiratory activation of the internal intercostal (*EMG_*I*_, ∫EMG_*I*_, II*) and oblique abdominal muscles (*EMG_*Abd*_, ∫EMG_*Abd*_*) during hyperpnea epochs (i,ii). Note that the epochs of hyperpnea initiate and terminate by an active expiration (arrows). **(B)** Sample trace of ventilatory motion and averaged signal synchronized with the first active expiration during hyperpnea epochs (i,ii, arrows; *N* = 30 epochs) showing that the function of active expiration is to force exhalation and increase the following inhalation.

**FIGURE 3:**
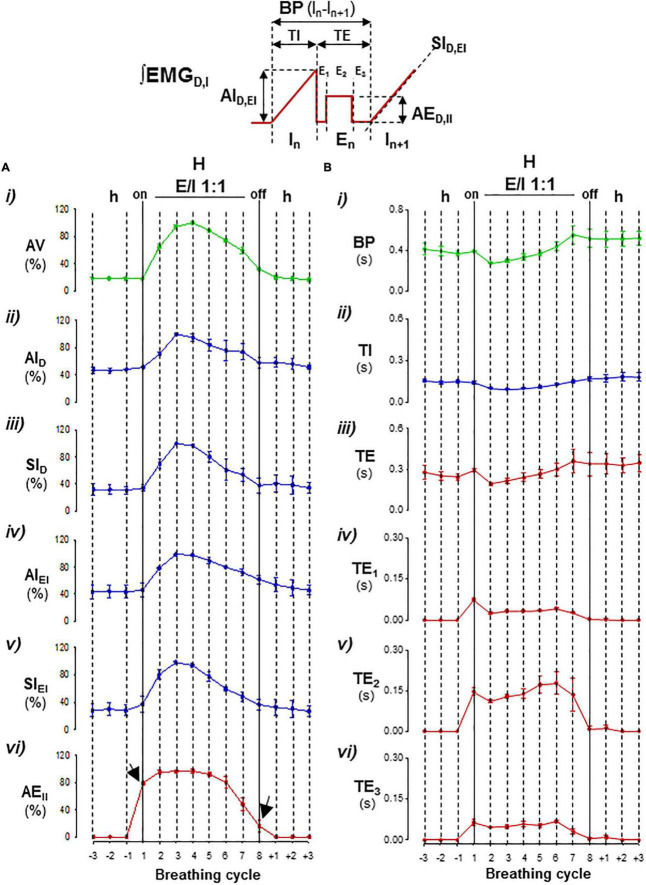
Parameterization cycle-by-cycle of expiratory–inspiratory coupling. *Inset*, diagram of respiratory parameters: *BP*, period of breathing cycle; *TI*, time of inspiration; *TE*, time of expiration with a passive postinspiratory phase (*TE*_1_), an active phase (*TE*_2_), and a passive preinspiratory phase (*TE*_3_); *IA* and *SI*, peak-intensity and slope of the inspiratory ramp of the diaphragm (*D*) and external intercostal muscles (*EI*), and *AE*_*II*_, peak-intensity of expiratory internal intercostal muscle. Mean values ± SEM from 15 hyperpnea epochs of eight breathing cycles/mouse; *N* = 5. **(A)** Graphs of mean motor intensities and slopes normalized relative to the maximum in each mouse; (i) ventilatory motion amplitude, *VA*, (ii–v) inspiratory activity, *IA_*D*_, SI_*D*_, IA_*EI*_, SI_*EI*_*, and (vi) expiratory intensity, *EA*_*II*_, versus the breathing cycle. **(B)** Graphs of mean durations of *BP* (i), *TI* (ii), and *TE, TE_1_, TE_2_*, and *TE*_3_ (iii–vi) versus the breathing cycle. Hypopnea–hyperpnea transitions and vice versa initiated and terminated by an active expiration (arrows); during expiratory–inspiratory coupling (*E/I 1:1*), the magnitude of *AV, IA_*D*_, SI_*D*_, IA_*EI*_, SI_*EI*_*, and *EA*_*II*_ values varied cycle-by-cycle in a crescendo–decrescendo manner (in **A**), while, in minus extension, the durations of *BP, TI, TE, TE_1_, TE_2_, and TE_3_* first shortened and then lengthened (in **B**).

### Statistical analysis

Averaged signals were represented with a 95% confidence band and the respiratory parameters as mean ± SEM. The relationships among respiratory parameters were evaluated with Pearson’s correlation coefficient (*R*) and a null hypothesis test with a significance level of *P* < 0.05.

## Results

### Neurogenic mechanism of periodic Cheyne–Stokes breathing

The periodic breathing previously identified in a subset of neonatal male Cx36KO mice (Pérez-Atencio et al., 2021; Supplementary Video 1) is present from the first up to the third week of life and then it disappears (Supplementary Figure 1). The comparison of this periodic respiratory pattern with the CSB of humans revealed that the periodic breathing of the Cx36KO mouse shares all pathognomonic features defining CSB, such as the rhythmic alternation of apnea/hypopnea and hyperpnea and crescendo–decrescendo modulation of ventilation and respiratory frequency, but on a ∼5-fold faster time scale and longer lasting hypopneas between hyperpnea epochs (Figures 1A, B), validating our mouse model for elucidating the neurogenic mechanisms of CSB.

The respiratory motor pattern of CBS in the Cx36KO mice was obtained by recording the EMG activity of principal and accessory inspiratory and expiratory muscles. EMG records showed a recurrent reconfiguration of the respiratory motor pattern between the epochs of hypopnea and hyperpnea. The hypopnea epochs are commanded by an exclusively inspiratory-driven rhythm of low-intensity synchronous in the diaphragm and the external intercostal muscles. On the other hand, the hyperpnea epochs are driven by a mixed inspiratory and expiratory pattern due to additional synchronous activation of the abdominal and internal intercostal expiratory muscles, giving rise to an expiratory–inspiratory coupling based on a 1:1 antiphase relationship (Figure 2A i, ii). Notice that the epochs of hyperpnea always started with an active expiration, driven by expiratory oscillator (Mellen et al., 2003; Onimaru and Homma, 2003; Janczewski and Feldman, 2006; Abdala et al., 2009; Huckstepp et al., 2015), and ended when this activity ceased, indicating that the hypopnea–hyperpnea alternation in CSB results from cyclic turn on/off of expiratory oscillator. The function of active expirations was to force the exhalation and enhance the subsequent inhalation to increase ventilation (Figure 2B i, ii; Pagliardini et al., 2011; Leirão et al., 2018). Expiratory–inspiratory coupling during hyperpnea epochs contained a relatively fixed number of breathing cycles (8.2 ± 2.4; *N* = 5 and 20 epochs/mouse) with a remarkable reproducibility of respiratory parameters cycle-by-cycle. The turn-on and -off of active expiration in the transitions from hypopnea to hyperpnea and vice versa provoked an abrupt jump in the peak intensity and slope of inspiratory motor ramps and ventilation amplitude (Figures 3A i–vi, 4A i, ii, arrows). Throughout expiratory–inspiratory coupling, the expiratory motor activity first increased and then decreased, and was always followed by the corresponding increase or decrease of intensity and slope of the inspiratory ramps (Figures 3A i–vi, 4A iii–v), which account for the crescendo–decrescendo modulation of ventilation. Furthermore, the frequency of respiratory rhythm during expiratory–inspiratory coupling also varied but in a smaller magnitude than the ventilatory amplitude; the duration of expiratory and inspiratory motor activities and the phase of active expiration occurrence into the breathing cycle first shortened and then lengthened causing the acceleration-deceleration of respiratory frequency during hyperpnea epochs (Figures 3B i–vi, 4B i–iv). Thus, the expiratory oscillator, in addition to triggering expiratory activity, entrains the inspiratory oscillator in a crescendo–decrescendo manner acting as the master pacemaker of CSB.

**FIGURE 4:**
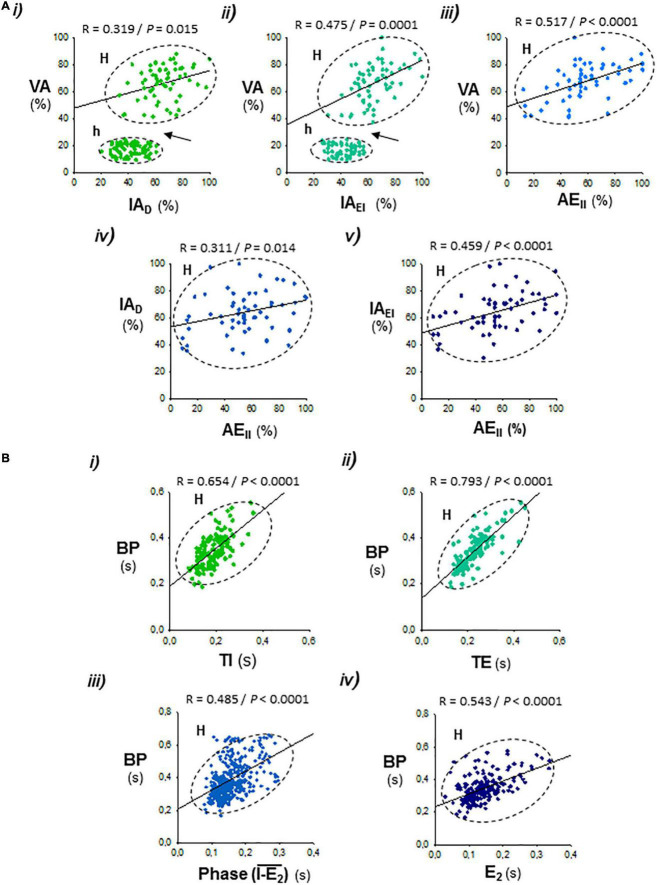
Correlations between the respiratory parameters during expiratory–inspiratory coupling. Data set from Figure 3. **(A)** Positive correlations of the peak intensity of inspiratory and expiratory activities of the diaphragm and external and internal intercostal muscles (*AI_*D*_, AI_*EI*_*, and *AE*_*II*_) with the amplitude of ventilation (*AV*; i–iii), and the peak intensity of expiratory internal intercostal activity (*AE*_*II*_) with the inspiratory diaphragm and external intercostal activities (*AI_*D*_, AI_*EI*_*; iv, v); note the abrupt jump of *AV* in the hypopnea–hyperpnea transitions (*h* and *H*, arrows in i and ii). **(B)** Period of the breathing cycle (*BP*) correlates positively with the inspiration and expiration time (*TI, TE*; i, ii), the phase of occurrence of active expiration within the respiratory cycle, defined by the interval between the starting of the inspiratory ramp and the beginning of active expiration (*I-E_2_*; iii), and with the duration of active expiration (*TE*_2_; iv). *R*, Pearson’s correlation coefficient; *P*, level of statistical significance.

### CO_2_ regulation of periodic Cheyne–Stokes breathing

**FIGURE 5:**
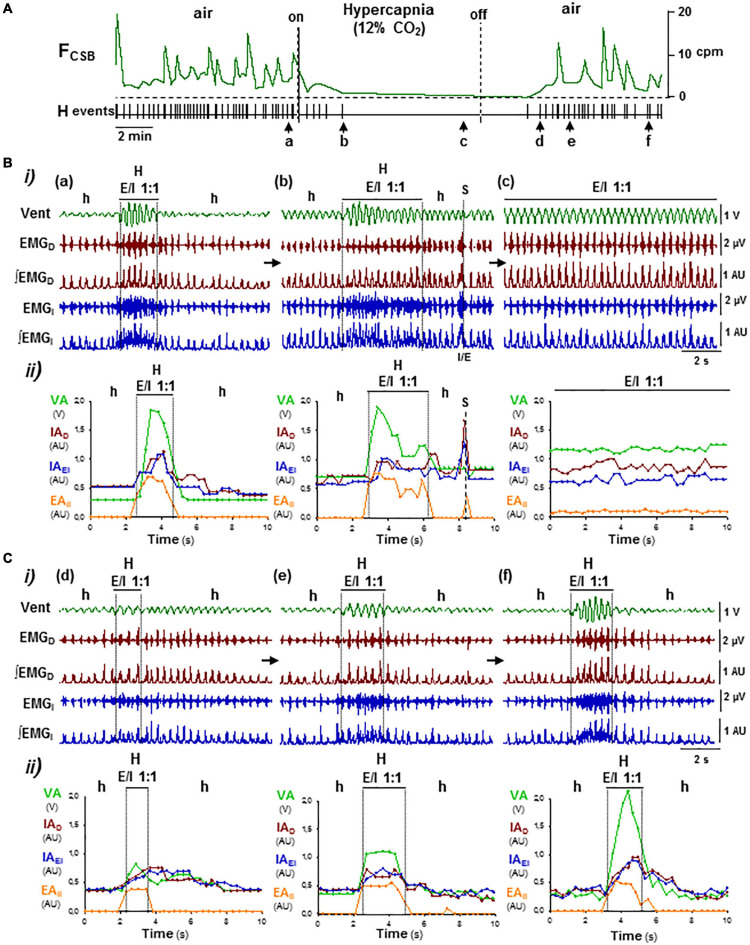
OFF/ON reconfigurations of CSB motor pattern induced by hypercapnia–normoxia. **(A)** Supplemental administration of CO_2_ and its removal in inhaled air of Cx36KO mice (12% CO_2_ in normoxia) suppressed reversibly CSB (*on/off*); *F*_*CSB*_, oscillatory frequency of CSB and *HP*, point process of hyperpnea epochs. Arrows **(a–f)**, changes in the respiratory pattern shown in **(B,C)**. **(B,C)** Off and on reconfigurations of the respiratory motor pattern leading to suppression (**a–c** in **B**) and reboot of CSB (**d–f** in **C**). (i) *Vent*, ventilatory motion; *EMG_*D*_, ∫EMG_*D*_, EMG_*I*_*, and *∫EMG_*I*_*, raw and rectified inspiratory and expiratory activities of the diaphragm (*D*) and external and internal intercostals (*EI, II*), and (ii) superimposed graphs cycle-by-cycle of the ventilatory amplitude (*VA*), peak-intensity of the inspiratory diaphragm and the external intercostal (*IA*_*D*_ and *IA*_*EI*_) and expiratory internal intercostal (*EA*_*II*_) activities versus. time; *h/a* and *H*, hypopnea/apnea and hyperpnea epochs; *S*, sigh. High CO_2_ regularized breathing by turning the expiratory–inspiratory coupling from cyclic to continuous (*E/I 1:1*, **a–c**; see detail in Figure 6). Washout of CO_2_ excess first, halted expiratory activity and depressed inspiratory activity and ventilation **(c,d)** and then rebooted CSB by the reactivation of expiratory activity and cyclic expiratory/inspiratory coupling **(d–f)**.

**FIGURE 6:**
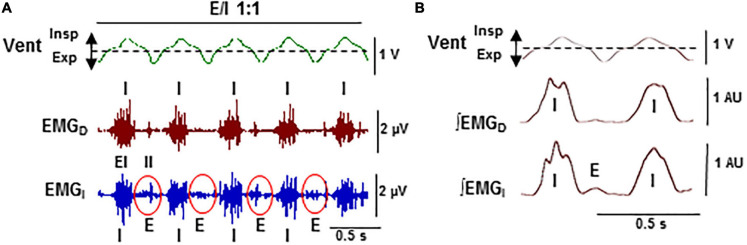
Stabilization of expiratory–inspiratory coupling in high CO_2_ causes the suppression of Cheyne–Stoke breathing. Detail of Figure 5B-c. **(A)** Records of the ventilatory motion (*Vent*; *Insp* and *Exp*, inspiration and expiration) and raw EMG of the diaphragm and intercostal muscles (*EMG_*D*_, EMG_*I*_*; *EI* and *II*, external and internal intercostals). **(B)** Average of ventilation signal (*Vent*) and fully rectified EMG signals (*∫EMGD, ∫EMGI*; *N* = 20 breathing cycles). Note the stable 1:1 alternation in antiphase (*E/I 1:1*) of the synchronous inspiratory activities of the diaphragm and the external intercostal muscles (*I*) with the activity of expiratory internal intercostal muscles (*E*).

Supplemental CO_2_ administration in the inhaled air (12% CO_2_ in normoxia) of the Cx36KO mice increased partial pressure of arterial CO_2_ (PtcCO_2_) from 48 + 5 to 69 + 7 mmHg (*N* = 5), suppressed CSB, and regularized respiration (Figure 5A), as occurring in human beings with CSB (Berssenbrugge et al., 1983; Xie et al., 1997; Giannoni et al., 2010; Sands et al., 2016). Lower CO_2_ concentrations were inefficient (4%) or did not regularize the respiration completely (8%). High CO_2_ abolished CSB through a gradual reconfiguration of respiratory motor pattern during the next 4–7 CSB cycles from stimulus onset. Initially, mice responded to the increment of CO_2_ with an enhancement of inspiratory activity and ventilation during the epochs of hypopnea, and an increase of the number of breathing cycles that the expiratory/inspiratory coupling lasts during the hyperpnea epochs until the hypopnea–hyperpnea alternation disappeared completely because the 1:1 expiratory–inspiratory entrainment became continuous (Figures 5B a–c, 6). The regularization of breathing improved ventilatory rate, but the exposition to an elevated CO_2_ produced a rapid compensatory decrease in the intensity of expiratory and inspiratory activities and ventilation concerning their peak values during the epochs of hyperventilation of CSB. This stable expiratory–inspiratory pattern is indistinguishable from that induced by hypercapnia in wild-type mice (Pérez-Atencio et al., 2021). During the washout of CO_2_ excess, CSB rebooted. First, active expiration disappeared, inspiratory activity decreased, and the animal fell again into hypopnea. Next, when ventilation depression was more marked, typically below the 20–30% ventilation rate induced by hypercapnia, hyperpnea epochs restarted gradually by a progressive reactivation of active expiration and 1:1 expiratory/inspiratory coupling; simultaneously, the inspiratory activity and ventilation further decreased during the hypopnea epochs (Figures 5C d–f). It indicates that CSB emerges as a mechanism of “rescue” in response to the disability of the inspiratory oscillator to sustain ventilation.

**FIGURE 7:**
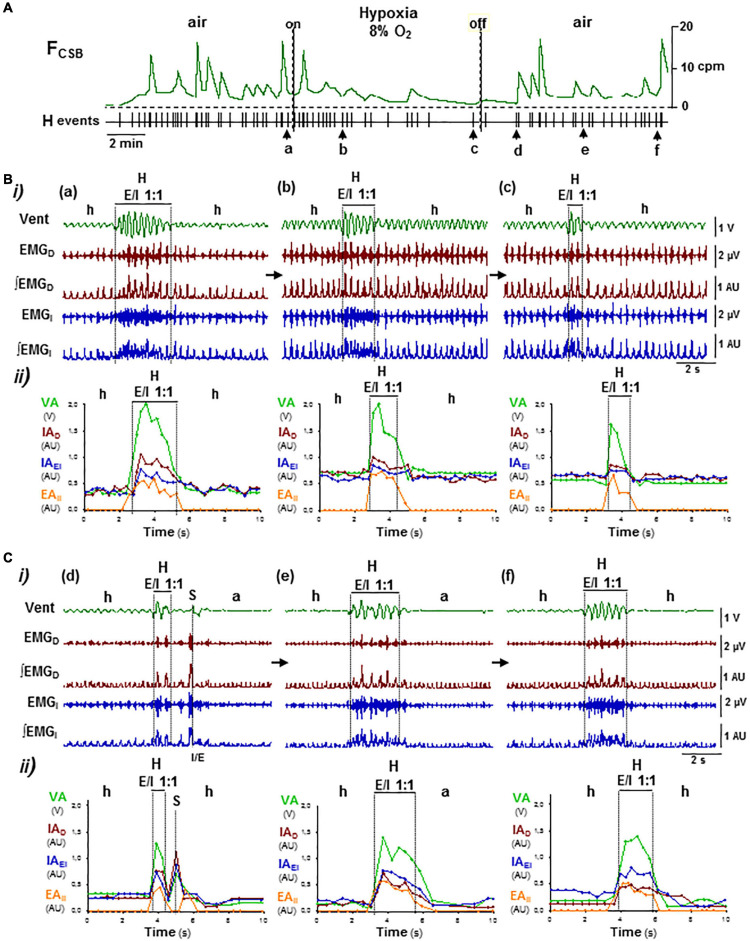
Reconfiguration of CSB motor pattern induced by hypoxia–hypocapnia. Symbols as in Figure 5. **(A)** Lowering O_2_ content in the inhaled air from 21 to 8% did not suppress CSB but slowed its oscillatory frequency (FCSB). **(B,C)** Reconfigurations of CSB motor pattern during hypoxia stimulus (**a–c** in **B**) and reoxygenation (**d–f** in **C**). Hypoxic stimuli first increased and then decreased inspiratory motor activity and ventilation during the epochs of hypopnea and hyperpnea epochs, reducing the number of breathing cycles that expiratory/inspiratory coupling lasts while the profile of motor activities changed from crescendo–decrescendo to only decrescendo **(a–c)**. Reoxygenation provoked a long-lasting depression of inspiratory activity and ventilation during the epochs of apnea/hypopnea while expiratory–inspiratory coupling during hyperpnea epochs recovered **(d–f)**.

**FIGURE 8:**
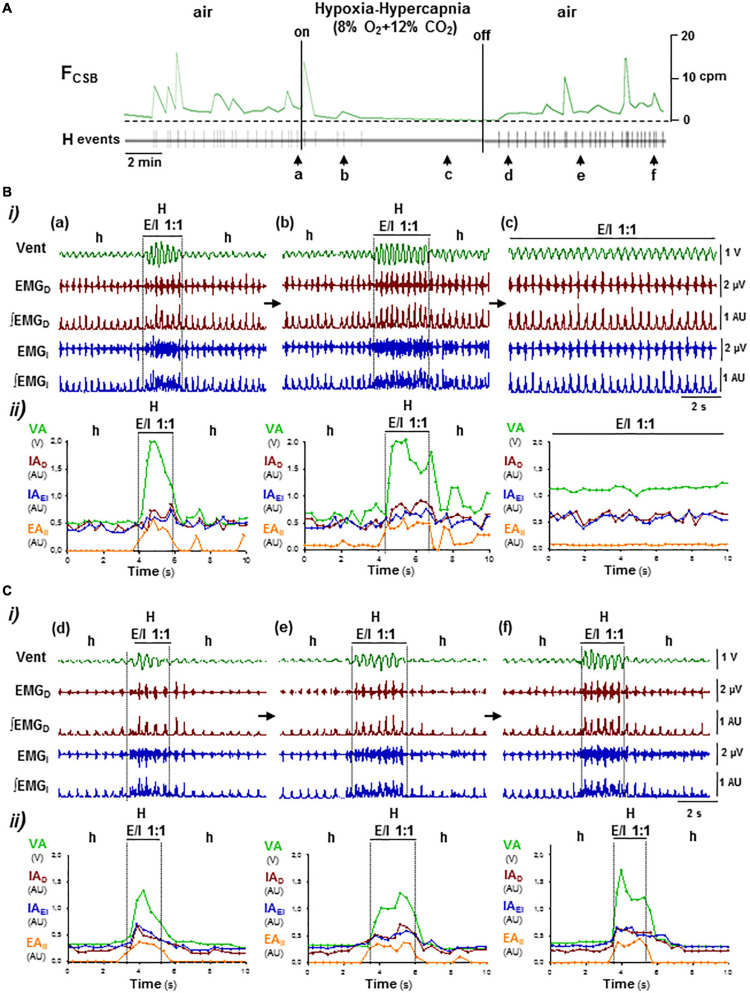
OFF/ON reconfigurations of CSB motor pattern induced by hypoxia–hypercapnia. Symbols as in Figure 5. **(A)** Administration and removal of low O_2_ (8%) with high CO_2_ (12%) in inhaled air suppressed and rebooted CSB, respectively. **(B,C)** Off and on reconfigurations of the respiratory motor pattern leading to suppression (**a–c** in **B**) and reboot of CSB (**d–f** in **C**). Hypoxia–hypercapnia stimulus enhanced and regularized breathing by turning expiratory/inspiratory coupling from cyclic to continuous (*E/I 1:1*, **a–c**). The reintroduction of medical air abolished expiratory activity and caused a profound depression of inspiratory activity and ventilation over which CSB rebooted gradually **(d–f)**.

Lowering O_2_ concentration in inhaled air from 21 to 8% caused a rapid desaturation of arterial O_2_ (SaO_2_) from 97 ± 2 to 48 ± 5% and secondarily reduced PtcCO_2_ from 48 + 4 to 28 + 9 mmHg (*N* = 5; Basting et al., 2015), which slowed down oscillatory period of CSB but did not suppress it (Figure 7A). Mice responded to hypoxia–hypocapnia with a transient increase in the intensity of inspiratory activity and ventilation rate during the hypopnea epochs and during the hyperventilation epochs, with a progressive reduction of the breathing cycles that 1:1 expiratory/inspiratory entrainment lasts, typically from 6–10 to only 2–3 cycles, while the profile of expiratory and inspiratory motor activities changed from crescendo–decrescendo to solely decrescendo (Figures 7B a–c). Reoxygenation at the end of hypoxic stimuli caused a long-lasting depression of inspiratory activity and ventilation during the epochs of hypopnea, while the duration and crescendo–decrescendo profile of expiratory and inspiratory activities recovered gradually (Figures 7C d–f). Stimuli of hypoxia in combination with hypercapnia (8% O_2_ and 12% CO_2_) that reduced SaO_2_ to 34 ± 4% and increased P_*tc*_CO_2_ to 78 ± 6 mmHg (*N* = 5) suppressed and rebooted CSB through similar off/on reconfigurations of the motor pattern than stimuli of hypercapnia in normoxia did (Figures 8A, B a–c, C d–f), indicating that the effect of hypoxia stimuli on expiratory/inspiratory entrainment should attribute to the hypocapnia secondary to hypoxia rather than hypoxia by itself.

## Discussion

The neurogenic mechanism of CSB identified here unveils that the incapacity of the inspiratory oscillator to drive ventilation is the triggering factor of CSB and that under these circumstances, the expiratory oscillator takes over respiration to overcome apnea/hypopnea. The activation of the expiratory oscillator throughout a CO_2_-dependent mechanism provokes hyperventilation by changing the mode of expiration from passive to active and entraining to the inspiratory oscillator. Given that the periodic breathing of the neonatal Cx36KO mouse shares all features of CSB in humans, this mechanism may be of general application for most types of periodic breathing, independently of etiology. On the contrary, this study reveals that Cx36 deletion increases the incidence at the postnatal period of CSB that later disappears during posterior maturation, as occurs in the preterm and term infants with CSB (Fenner et al., 1973), indicating that the high level of Cx36 expression during the first weeks of life (Belluardo et al., 2000; Solomon, 2003) protects against CSB. Cx36 is considered the major component of electrical synapses in the CNS due to its widespread pattern of expression in multiple structures (Deans et al., 2001). At the brainstem, Cx36 expresses in those nuclei involved in the generation of respiratory rhythm, as preBötzinger and Bötzinger complexes (preBötC and BötC), and central chemoreception, as the retrotrapezoidal nucleus (RTN), the solitary tract nucleus, the dorsal raphe nucleus, and the locus coeruleus (Belluardo et al., 2000; Solomon, 2003). Indeed, in a previous study, we found that mice lacking Cx36 at P14 exhibited respiratory instability and CO_2_-exacerbated chemoreflexes (Pérez-Atencio et al., 2021). Consistent with the “loop gain” theory of CO_2_ feedback control (Khoo et al., 1982; Sands et al., 2016), this increased sensitivity to CO_2_ predicts the elevated propensity of the Cx36KO mouse to develop CSB.

**FIGURE 9:**
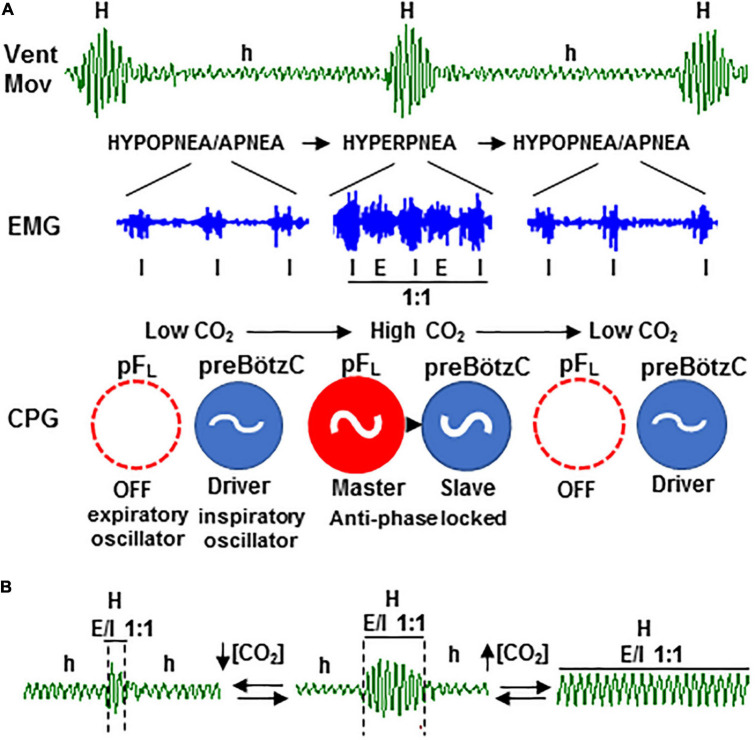
Rhythmogenic mechanism of periodic Cheyne–Stokes breathing based on the two-oscillator system. **(A)** Self-sustained hypopnea-hyperpnea alternation in CSB (*Vent*; *h* and *H*) results from the recurrent reconfiguration of the respiratory motor pattern (*EMG*) between an inspiratory-driven rhythm of low motor activity (*I*) during the hypopnea epochs and an expiratory and inspiratory rhythm coupled in a ratio of 1:1 (*E/I* 1:1) during hyperpnea epochs, denoting the cyclic turn on/off of the expiratory pF_*L*_ oscillator of central pattern generator (*CPG*, in red); expiratory oscillator acts as an “anti-apnea” pacemaker that entrains inspiratory preBötzC oscillator (in blue) for rescuing its disability to sustain ventilation. **(B)** Coupling between the expiratory and inspiratory oscillators is CO_2_-dependent; *PaCO*_2_ increase above the apneic threshold suppresses CSB by stabilizing expiratory–inspiratory coupling (right) while *PaCO*_2_ reduction diminishes to the minimum the number of breathing cycles that expiratory–inspiratory coupling lasts (left).

The novel neurogenic mechanism described here provides fundamental insights concerning the genesis and therapy of CSB. The periodic reconfiguration of respiratory motor pattern between hypopnea and hyperpnea results from the cyclic turn on/off of the expiratory oscillator, which acts as a master pacemaker of respiration and entrains inspiratory oscillator to rescue ventilation (Figure 9A); the cyclic turn on/off of the expiratory oscillator is the result and source of self-sustained CO_2_ oscillations around the apneic threshold responsible for CSB (Berssenbrugge et al., 1983). Thus, the active expiration driven by the expiratory oscillator when CO_2_ rises during the epochs of hypopnea provokes an exacerbated ventilation or hyperpnea which, in turn, creates a CO_2_ imbalance in the opposite direction that silences the expiratory oscillator, and therefore, the animal falls again in hypopnea, and the whole cycle repeats. Consequently, the apneic threshold in periodic breathing is functionally linked to activation/inactivation of the expiratory oscillator since the exit and re-entry in apnea/hypopnea are triggered by the transitions of expiratory mode from passive to active and vice versa. To this respect, the all-or-none nature of transitions and the subsequent abrupt change in the inspiratory activity and ventilation rate introduces an outstanding non-linearity in the “loop gain” theory for the feedback system controlling CO_2_ (Khoo et al., 1982), which can explain the abrupt onset in ventilation after apnea and the propensity to CSB (Wilkinson et al., 1996). Those hyperpnea epochs of CSB initiated and terminated by an active expiration reflect prompt responsiveness of the expiratory oscillator to CO_2_; however, the mechanism involved in the recruitment of the expiratory oscillator by hypercapnia is not fully understood. The rhythmogenic neurons of the expiratory pF_*L*_ oscillator expressing Phox2b transcription factor in neonatal rats are intrinsically CO_2_-sensitive (Onimaru et al., 2008); however, it is unknown whether this intrinsic sensitivity to CO_2_ persists in the pF_*L*_ neurons of juvenile/adult rats with undetectable levels of Phox2b (De Britto and Moraes, 2017). Alternatively, the activation of pF_*L*_ neurons of the expiratory oscillator may be determined by the balance of excitatory and inhibitory synaptic inputs from other respiratory compartments. Excitatory inputs from the CO_2_-sensitive Phox2b neurons of the retrotrapezoidal nucleus (RTN, also termed ventral parafacial group, pFv), which acts as a nodal point for integrating afferents from peripheral chemoreceptors and other central chemosensitive sites, seem necessary for the activation of pF_*L*_ neurons and expiratory musculature during hypercapnia (Marina et al., 2010; Abbott et al., 2011; Burke et al., 2015; Huckstepp et al., 2015; Zoccal et al., 2018). Other studies indicate that the pF_*L*_ neurons of the expiratory oscillator are synaptically inhibited at normocapnia and activate during hypercapnia by a mechanism of disinhibition (Pagliardini et al., 2011; De Britto and Moraes, 2017); to this respect, inhibitory projections from the Bötzinger complex (BötC) to pF_*L*_ seem to be an essential part of the neural circuitry controlling the generation of active expiration (Flor et al., 2020).

When active expiration is triggered during the hyperpnea epochs of CSB, expiratory and inspiratory motor activities synchronize through an antiphase-locked mechanism in a ratio of 1:1, denoting the entrainment of the inspiratory oscillator by the expiratory oscillator. This coupling is dynamically modulated cycle-by-cycle mainly in amplitude and, in less magnitude, in frequency which explains the crescendo–decrescendo enhancement of ventilatory amplitude and respiratory frequency during the hyperpnea epochs. In this context, the greater amplitude of end-expiratory lung volume during hyperpnea epochs of CSB reported in patients with heart failure and worse cardiac function may be interpreted as an exacerbated expiratory muscle recruitment (Perger et al., 2017). The interaction between oscillators relies primarily on specific reciprocal synaptic interactions between pF_*L*_ neurons and preBöt/BötC neurons (Tan et al., 2010; Huckstepp et al., 2016; Biancardi et al., 2020; Flor et al., 2020) and is probably also coordinated by their specific feedback afferents from peripheral chemoreceptors and pulmonary stretch receptors (Janczewski and Feldman, 2006; Molkov et al., 2014). Thus, the expiratory oscillator, in addition to driving the active expiratory rhythm *via* exciting expiratory premotor neurons (Janczewski et al., 2002), acts in CSB as a master pacemaker that entrains an inspiratory oscillator for driving ventilation. The hierarchical organization between the two respiratory oscillators during CSB contrasts with the paradigm of a principal inspiratory oscillator and a subsidiary conditional expiratory oscillator driving the eupneic respiration (Janczewski and Feldman, 2006; Pagliardini et al., 2011; Huckstepp et al., 2015, 2016). In this regard, data of nascent CSB following its suppression by high CO_2_ and washout of CO_2_ excess show that CSB only reboots when the inspiratory activity is profoundly depressed, indicating that the expiratory oscillator only paces breathing when the driving force of the inspiratory oscillator is seriously compromised. Experiments of pharmacogenetic hyperpolarization of preBötC neurons also show that when the inspiratory activity decreases significantly, the expiration becomes active and respiration unstable alternating epochs of apnea and breathing (Huckstepp et al., 2016). In the case of the Cx36KO mouse, the less robustness of the inspiratory drive may be due to the loss of electrical coupling and subsequent desynchronization of rhythmogenic preBötC neurons (Rekling et al., 2000). Thus, CSB emerges as an adaptive mechanism in which the expiratory oscillator acts as an “anti-apnea” center capable of rescuing the inspiratory oscillator activity to sustain ventilation. Consequently, the dysfunction of the inspiratory oscillator might be considered the triggering factor in the genesis of CSB. The rescue function of the expiratory oscillator has been previously proposed for overcoming the selective depression of the inspiratory oscillator induced by opioids (Takeda et al., 2001; Janczewski and Feldman, 2006; Oku et al., 2007) or when respiration is irregular like during REM epochs to improve respiratory stability and ventilation (Andrews and Pagliardini, 2015).

The stability of expiratory–inspiratory entrainment during hyperpnea epochs of CSB depends on the CO_2_ level, increasing with hypercapnia and decreasing with hypopnea (Figure 9B). Similar to healthy subjects under hypobaric hypoxia and patients with CSB (Xie et al., 1997; Giannoni et al., 2010; Sands et al., 2016), inhalation of a CO_2_-enriched gas mixture to elevate the CO_2_ level above the apneic threshold abolishes the periodic respiration in our animal model with CSB. Notice that a CO_2_ concentration below 2% is enough to suppress CSB in patients (Sands et al., 2016), but the mouse required much higher concentrations (8–12% CO_2_). In this regard, our data indicate that the neural mechanism by which supplemental CO_2_ regularizes breathing is by stabilizing the coupling between expiratory and inspiratory oscillators, providing a rationale neural base for the therapeutic use of supplemental CO_2_ in CSB patients. However, the continuous administration of CO_2_ causes undesirable elevations in partial pressure of CO_2_, mean ventilation, and sympathetic activity, limiting its clinical applicability (Wan et al., 2013). The real-time dynamic administration, which allows CO_2_ delivery during a small fraction of the CSB cycle, is equally efficient for switching off CSB than static administration but avoids mostly undesirable effects (Sands et al., 2016). Theoretical and clinical studies of dynamic CO_2_ delivery found that the optimal phase window is between −30° and +40° around the peak of hyperventilation (0°) and that the delivery outside of this phase range further increases CSB oscillations (Mebrate et al., 2009; Giannoni et al., 2010). In light of these findings, we postulate based on our new model of CSB driven by the CO_2_-dependent activation of the expiratory oscillator that the CO_2_ delivery at the optimal phase would counteract the transient reduction in partial pressure of CO_2_ preventing the silencing of the expiratory oscillator and the subsequent hypoventilation. In contrast, the CO_2_ delivery outside the optimal phase would augment hypercapnia and overstimulate the expiratory oscillator causing exaggerated hyperventilation.

## Author’s note

We currently know that the respiratory rhythm arises from the concerted action in the brainstem of two generators pacing the rhythm of the contraction of the inspiratory and expiratory musculature of the respiratory pump. However, a fundamental question unresolved is how this system of two oscillators generates respiratory rhythm disturbances. Periodic breathing characterized by the recurrent alternation of apnea and hyperventilation is the most common central apnea for which there is no proven therapy because its neural mechanism of generation is unknown. Here, we report that periodic breathing results from the cyclic turn on/off of active expiration driven by the expiratory oscillator, which also entrains the inspiratory oscillator to restore ventilation. Our results also show that the stabilization of the coupling between the expiratory and inspiratory oscillator causes the suppression of periodic breathing and the regularization of breathing induced by supplemental administration of carbon dioxide. The neural mechanism of periodic breathing identified reveals that the triggering factor of apnea/hypopnea is the inability of the inspiratory oscillator to sustain ventilation and that, under these circumstances, the expiratory oscillator activated by the increase in carbon dioxide acts as an “anti-apnea” center. This mechanism highlights the plasticity of the two-oscillator system in the neural control of respiration and provides the pathophysiological basis for carbon dioxide therapy of periodic respiration.

## Data availability statement

The original contributions presented in this study are included in the article/Supplementary material, further inquiries can be directed to the corresponding author.

## Ethics statement

The Local Ethics Committee approved animal handling and experimentation protocols (PROEX 165/16), according to the application of European 86/609 and Spanish 1201/2005 laws.

## Author contributions

LB designed the study and wrote the manuscript. DP, JI, and EM contributed respectively with a knockout mouse model, new devices, and patients’ data. AC, LP-A, and CM performed the experiments, analyzed the data, and revised the manuscript. All authors contributed to the article and approved the submitted version.
