# Supplementary material for: Neural bases for the genesis and CO2 therapy of periodic Cheyne–Stokes breathing in neonatal male connexin-36 knockout mice
Source: Front Neurosci. 2023 Feb 8;17:1045269. doi: 10.3389/fnins.2023.1045269 (PMC9944137; doi:10.3389/fnins.2023.1045269)
Supplement: Supplementary file 1 [file Data_Sheet_1.PDF]

SUPPLEMENTARY MATERIAL

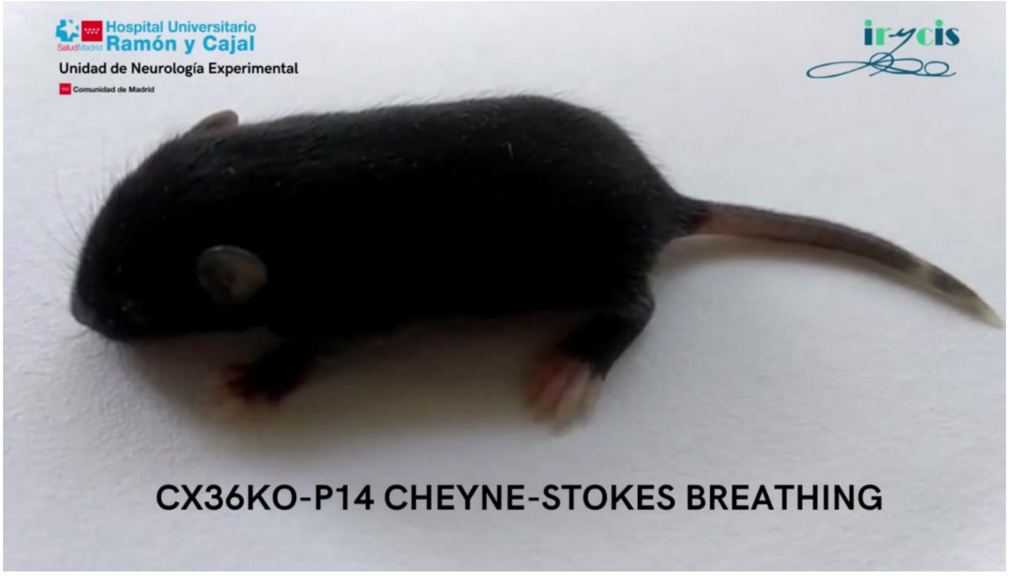

Video 1. Postnatal Cx36-knockout mouse (P14) with periodic Cheyne-Stokes breathing. Mouse breathing alternates periodically between hypopnea and hyperpnea all the time, at resting and during exploratory behaviour (Cx36KO_P14_CSB(1).avi).

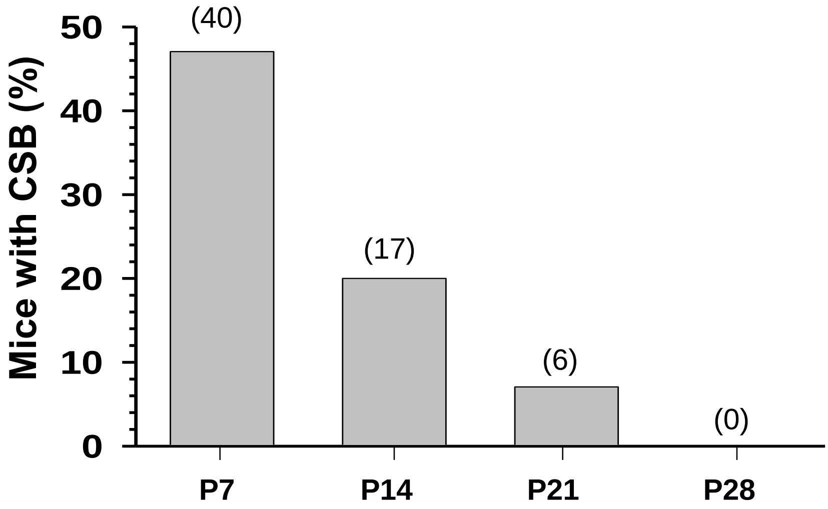

Supplementary Figure 1. Temporal window of period breathing in the male Cx36-knockout mice at postnatal period. Percentage of male mice with periodic breathing vs. total male neonates (*N*=85) decreases from 1st to 4th week of postnatal life until it disappears.
